# Larger Amygdala Volume Mediates the Association Between Prenatal Maternal Stress and Higher Levels of Externalizing Behaviors: Sex Specific Effects in Project Ice Storm

**DOI:** 10.3389/fnhum.2019.00144

**Published:** 2019-05-14

**Authors:** Sherri Lee Jones, Romane Dufoix, David P. Laplante, Guillaume Elgbeili, Raihaan Patel, M. Mallar Chakravarty, Suzanne King, Jens C. Pruessner

**Affiliations:** ^1^Laboratory of Suzanne King, Department of Psychiatry, Douglas Mental Health University Institute, McGill University, Montreal, QC, Canada; ^2^Laboratory of Suzanne King, Douglas Mental Health University Institute, Montreal, QC, Canada; ^3^Computational Brain Anatomy Laboratory (CoBrA Lab), Douglas Mental Health University Institute, Montreal, QC, Canada; ^4^Cerebral Imaging Center, Douglas Mental Health University Institute, Montreal, QC, Canada; ^5^Department of Biological and Biomedical Engineering, McGill University, Montreal, QC, Canada; ^6^Laboratory of Jens Pruessner, Department of Psychology, University of Konstanz, Konstanz, Germany

**Keywords:** prenatal maternal stress, amygdala, internalizing, externalizing, adolescence

## Abstract

**Introduction:** The amygdala is a brain structure involved in emotional regulation. Studies have shown that larger amygdala volumes are associated with behavioral disorders. Prenatal maternal depression is associated with structural changes in the amygdala, which in turn, is predictive of an increase in behavioral problems. Girls may be particularly vulnerable. However, it is not known whether disaster-related prenatal maternal stress (PNMS), or which aspect of the maternal stress experience (i.e., objective hardship, subjective distress, and cognitive appraisal), influences amygdala volumes. Nor is it known whether amygdala volumes mediate the effect of PNMS on behavioral problems in girls and boys.

**Aims:** To assess whether aspects of PNMS are associated with amygdala volume, to determine whether timing of exposure moderates the effect, and to test whether amygdala volume mediates the association between PNMS and internalizing and externalizing problems in 11½ year old children exposed *in utero*, to varying levels of disaster-related PNMS.

**Methods:** Bilateral amygdala volumes (AGV) and total brain volume (TBV) were acquired using magnetic resonance imaging, from 35 boys and 33 girls whose mothers were pregnant during the January 1998 Quebec Ice Storm. The mothers' disaster-related stress was assessed in June 1998. Child internalizing and externalizing problems were assessed at 11½ years using the Child Behavior Checklist (CBCL). Hierarchical regression analyses and mediation analyses were conducted on boys and girls separately, controlling for perinatal and postnatal factors.

**Results:** In boys, subjective distress was associated with larger right AGV/TBV when mothers where exposed during late pregnancy, which in turn explained higher levels of externalizing behavior. However, when adjusting for postnatal factors, the effect was no longer significant. In girls, later gestational exposure to the ice storm was associated with larger AGV/TBV, but here, higher levels of objective PNMS were associated with more externalizing problems, which was, in part, mediated by larger AGV/TBV. No effects were detected on internalizing behaviors.

**Conclusion:** These results suggest that the effects of PNMS on amygdala development and externalizing symptoms, as assessed in boys and girls in early adolescence, can be influenced by the timing of the stress in pregnancy, and the particular aspect of the mother's stress experience.

## Introduction

There is strong evidence that prenatal maternal stress (PNMS) or anxiety during pregnancy has profound and long-lasting adverse consequences on offspring development (see Van den Bergh et al., 2017 for a recent review). For example, pregnancy-specific anxiety has been associated with increased adolescent depression levels in the offspring (Van Lieshout and Boylan, 2010; Van den Bergh et al., 2017) and an increase in externalizing problems in toddlers (Wadhwa et al., 2001; Gutteling et al., 2005; Chuang et al., 2011; Liu, 2011). As reviewed in Glover and Hill (2012), prospective studies worldwide show that mothers who are stressed, anxious, or depressed have children that are more likely to have symptoms of anxiety, depression, and increased stress reactivity. Some studies show that the associations are particularly sensitive to prenatal maternal anxiety (e.g., O'Connor et al., 2002; Van den Bergh and Marcoen, 2004). Many of those studies controlled for potential confounders associated with anxiety, such as smoking, alcohol use, and household income, and showed that the results persisted. Importantly, these effects even persist after controlling for genetic factors using prenatal cross-fostering studies (i.e., *in-vitro* fertilization) (Rice et al., 2010). Finally, Buss et al. (2012) reported that higher maternal cortisol levels during early pregnancy predicted more internalizing problems in girls at age 7, but not in boys, which was in part, mediated by amygdala volume (AGV).

The amygdala is an important limbic structure involved in processing of biologically relevant stimuli, emotional learning and memory (Davis and Whalen, 2001; Quirk and Beer, 2006), and is vulnerable to prenatal factors. Exposure to maternal depression has been shown to influence AGV and functionality. For example, chronic maternal depression is associated with increased AGV in 10 year old boys and girls (Lupien et al., 2011), and with deficits in amygdala connectivity and functionality at age 4½ years albeit only in girls (Soe et al., 2018). Prenatal exposure to maternal depression is associated with altered right amygdala microstructure at birth (Rifkin-Graboi et al., 2013), with AGV in childhood as well as with structural changes in the amygdala in the neonatal period and childhood (Rifkin-Graboi et al., 2013; Favaro et al., 2015; Posner et al., 2016; Scheinost et al., 2016; Wen et al., 2017). In a study considering maternal cortisol levels from saliva samples acquired at pregnancy weeks 15, 19, 25, 31, and 37, Buss et al. (2012) reported that exposure to higher levels of cortisol during earlier gestation was associated with larger right AGV in girls but not boys. Together these results suggest that the amygdalae of girls are particularly susceptible to the effects of maternal depression and pregnancy related cortisol levels.

Structural properties of the amygdala are also associated with psychopathology and subclinical psychopathological symptoms. For example, AGV is associated with psychiatric problems including depression (MacMillan et al., 2003; Rosso et al., 2005), anxiety disorders (De Bellis et al., 2000; Milham et al., 2005; Schienle et al., 2011), and aggression (Gopal et al., 2013), as well as sub-clinical symptoms of internalizing (e.g., anxiety and depression) (Spampinato et al., 2009) and externalizing (e.g., aggressive) behaviors (Matthies et al., 2012; Pardini et al., 2014; Thijssen et al., 2015).

The associations between perinatal stressors and/or naturally occurring maternal cortisol levels during pregnancy and AGV can explain psychopathological outcomes in the offspring. Interestingly, rodent studies suggest that PNMS may influence offspring development and subsequent behavioral outcomes by altering amygdala maturation as measured by AGV (Salm et al., 2004; Kraszpulski et al., 2009). Likewise, Buss et al. (2012) demonstrated that AGV mediated the relationship between maternal cortisol levels in early pregnancy and affective problems in 7 year-old girls, such that larger right AGV was associated with more affective problems. This finding is important as it shows that natural variations in cortisol during pregnancy can explain variation in AGV and in turn explain psychopathological symptoms, and highlights sex-specific effects as well as potential hemispheric lateralization.

While these findings link perinatal maternal depression and prenatal maternal cortisol to structural changes in the amygdala, and subsequent psychopathological symptoms in exposed offspring, these studies have their limitations. The use of maternal psychopathology as the stressor is confounded by potential genetic transmission from mother to child. Moreover, while the Buss et al. (2012) findings posit a potential mechanism—*in utero* exposure to heightened levels of maternal cortisol via the placenta (Cottrell and Seckl, 2009; Harris and Seckl, 2011)—maternal “stress” levels were not measured. Finally, although several studies suggest that the timing of a stressor *in utero* moderates the effects of maternal stress on childhood neurodevelopmental outcomes (Kinney et al., 2008; Cao et al., 2014; Moss et al., 2018), studies of maternal depression in pregnancy cannot usually identify a specific onset date. As such, the temporal link between exposure to perinatal stressors, AGV, and childhood psychopathology symptoms with a sensitivity to timing of exposure, remains unclear.

This gap can be addressed in our PNMS study of children exposed to a natural disaster just prior to, and during gestation: Project Ice Storm. In January 1998, a series of ice storms resulted in electrical power failures for more than 3 million individuals for periods varying from 6 h, to more than five weeks, during the coldest months of the year. Mothers of the youth in our prospective, longitudinal study were at various stages of pregnancy and were quasi-randomly exposed to varying degrees of storm-related hardship. The ice storm serves as a “natural experiment,” affecting women regardless of their personal characteristics while allowing for the women's disaster experience to be divided into various components (i.e., objective hardship exposure, cognitive appraisal of the event, and subjective distress from the event). As well, because the ice storm had a sudden onset, the timing of the onset of maternal stress can be assessed with great accuracy. Among the findings from Project Ice Storm, we have shown that increased objective hardship predicts delayed cognitive and language development in childhood (King and Laplante, 2005; Laplante et al., 2008), while both the women's objective hardship and subjective distress levels predict internalizing and externalizing problems in the children throughout childhood (King et al., 2012).

The goals of the present study were to test whether varying aspects of the PNMS experience affect AGV in 11½ year-old youth using a prospective longitudinal design, and whether AGV mediates the association between varying aspects of the PNMS experience and child internalizing and externalizing problems. We also hypothesized that the timing of the prenatal maternal stressor would moderate those associations.

## Materials and Methods

### Participants

This study was carried out in accordance with the recommendations of The Code of Ethics of the World Medical Association, and approved by the Douglas Mental Health University Institute Research Ethics Board. All subjects gave written informed consent or written informed assent for participants under the age of 18 years, in accordance with the Declaration of Helsinki.

The initial Project Ice Storm cohort consisted of 178 children whose mothers were pregnant during the 1998 Quebec ice storm or became pregnant within 3 months of the ice storm and who responded to the questionnaire “reaction to the storm” sent on June 1, 1998. At 11½ years, 100 families were approached about participating in a standardized assessment and a structural brain magnetic resonance imaging (MRI) study. Of those, 90 children (male = 47; female = 43) underwent the cognitive and behavioral assessment, and 71 agreed to undergo the MRI protocol (male = 35; female = 36). Two children (both female) refused to undergo the scanning upon seeing the scanner. The scan of one girl was unusable because of excessive movement. An additional five participants (male = 4; female = 1) who were born preterm (before 37 weeks) with a low birth weight were excluded from the analyses as these birth outcomes have been shown to affect brain structure and integrity (Buss et al., 2007). The mother of one male participant did not complete the behavioral questionnaire (CBCL), leaving a total of 62 (30 male, 32 female) participants with valid behavioral data, and a 63 participants (31 males, 32 females) with valid MRI data.

There were 2 left-handed boys, and 3 left-handed girls. Thirty-three percent (21/63) of the participants were exposed to the storm prior to conception (boys: 11/31; girls: 10/32), 22.2% (14/63) during the first trimester (boys: 7/31; girls: 7/32), 23.8% (15/63) during the second trimester (boys: 8/31; girls: 7/32) and 20.6% (13/63) during the third trimester (boys: 5/31; girls: 8/32).

### Instruments

#### Predictor Variables: Prenatal Maternal Stress (PNMS)

Objective hardship was assessed in June 1998 using Storm32 (Laplante et al., 2007), which assesses the mothers' responses to questions associated with four categories of exposure that were often used in other disaster studies: threat, loss, scope and change (Bromet and Dew, 1995). Scores for each category ranged from 0 to 8. A total score (max 32) was obtained by summing the categories.

Mothers' subjective distress was assessed in June 1998, using a validated French version (Brunet et al., 2003) of the 22-item Impact of Event Scale-Revised (IES-R; (Weiss and Marmar, 1997). This scale describes symptoms from three categories relevant to post-traumatic stress disorder (PTSD): hyper-arousal, avoidance, and intrusive thoughts or images. Thus, this scale reflects enduring PTSD symptoms in response to the ice storm crisis, which had begun 5 months earlier.

Mothers' cognitive appraisal of the ice storm crisis was assessed in the June 1998 questionnaire using the following question: “Overall, what were the consequences of the ice storm on you and your family?” Response options were on a 5-point Likert scale: “Very negative” (1), “Negative” (2), “Neutral” (3), “Positive” (4), and “Very positive” (5). This item was recoded into “negative” (0) or “neutral/positive” (1). We opted to treat cognitive appraisal as binary for two reasons. First, very few mothers reported that the crisis was either “very negative” (2) or “very positive” (1). Second, we wanted to isolate the “negative” appraisal from “neutral” and “positive” appraisals, because we believe negative cognitive appraisal is one of the components of the maternal stress experience that can influence child development.

Timing of *in utero* ice storm exposure was determined as the number of days between estimated date of conception and January 9th, 1998, the date at which the storm peaked; higher values indicate storm exposure later in pregnancy. To calculate estimated date of conception, 280 days (40 weeks) was subtracted from the women's due date.

Behavioral problems were assessed using the mother-rated 113-item Child Behavior Checklist (CBCL), the gold-standard for behavioral research, which yields scores on several subscales that combine to create Internalizing and Externalizing scales. The standardized scores with a mean of 50 and standard deviation of 10 were used in the analyses (Achenbach and Ruffle, 2000). Data for one boy were missing.

#### Control Variables

Socioeconomic status (SES) was measured with the Hollingshead scale (Hollingshead, 1975) using data from the June 1, 1998 (perinatal) and when the children were 11½ years of age (concurrent) questionnaires about maternal and paternal education and occupation. Note that the lower the score, the higher the SES. At recruitment, 1.6% (0/31 boys, 1/32 girls) were lower class, 3.2% (2/31 boys, 0/32 girls) were lower middle class, 31.7% (11/31 boys, 9/32 girls) were middle class, 46% (14/31 boys, 15/32 girls) were upper middle class, and 17.5% (4/31 boys, 7/32 girls) were upper class. At 11½ years, 3.4% (1/29 boys, 1/30 girls) were lower class, 11.9% (6/29 boys, 1/30 girls) were lower middle class, 30.5% (0/29 boys, 9/30 girls) were middle class, 32.2% (9/29 boys, 10/30 girls) were upper middle class, and 22.0% (4/29 boys, 9/30 girls) were upper class. Data for four families were missing.

The Life Experience Survey (LES) (Sarason et al., 1978) was used to control for any other major life events the mothers experienced during their pregnancies and between when the children were 2½ to 11½ years of age, The perinatal questionnaire was completed by the mothers when the children were 6 months of age. This questionnaire covered the period between conception and when the child was 6 months of age. The three postnatal questionnaires were completed by the mothers when the children were 5½, 8½, and 11½ years of age. Each questionnaire covered the previous 36 months. Perinatal data from one mother (girl) were missing. Data were missing from 29 mothers (14 boys, 15 girls) at 5½ years, 10 mothers (5 boys; 5 girls) at 8½ years, and one mother (boy) at 11½ years. In order to control for missing data, the average number of major events per year was calculated between ages 2½ and 11½.

Mothers' concurrent psychological symptoms were assessed when the children were aged 11½ using the General Health Questionnaire (GHQ-28; Goldberg, 1972). Each of its 28 items describes a psychological or somatic symptom, and subjects indicate on a 4-point Likert scale how much they have experienced it in the preceding 2 weeks. There are subscales for Anxiety, Depression, Somatic Complaints, and Dysfunction. In the present study, each item was rescored as either 0 (a rating of 0 or 1) or 1 (a rating of 2 or 3), according to the Goldberg method (Goldberg, 1972), resulting in a minimum possible score of 0 and a maximum possible score of 28. Data from two mothers (one girl, one boy) were missing.

The number of obstetric complications (e.g., hypertension, preeclampsia, cold or flu) was determined by maternal recall when their children were 6 months of age using an adaptation of the checklist used by Jacobsen and Kinney (1980) and, when available, by examination of medical charts. The total number of obstetric complications experienced by the women that were rated as moderate-to-severe using the McNeil-Sjöström Scale for Obstetric Complications (McNeil and Sjöström, 1995) were used in the analyses.

Smoking and alcohol habits in pregnancy were assessed in the 6 month postpartum questionnaire in order to capture the entire pregnancy. Women were asked to indicate the number of cigarettes smoked per day, and the number of drinks consumed per week.

Child handedness (e.g., right- or left-handed) was determined at age 11½ by observing which hand the child used to complete a visual-motor integration task.

### MRI Image Acquisition

Anatomical MRI was performed at the *Unité de Neuroimagerie Fonctionelle* (UNF) *du Center de Recherche de l'Institut Universitaire de Gériatrie de Montréal* (CRIUGM). Brain imaging was done with a 3.0T Siemens MAGNETOM Trio TIM Syngo magnetic resonance imaging (MRI) scanner (Siemens, Erlangen, Germany), with a 12-channel head coil. For each participant, we obtained a 3D, T1-weighted Magnetization Prepared Rapid Gradient Echo [MPRAGE] sequence (TR/TE/TI = 2300/2.98/900 ms) (voxel size = 1 × 1 × 1 mm^3^; sagittal acquisition; 176 slices; 256 × 256 mm grid). The raw images underwent automated correction for intensity non-uniformity and normalization for signal intensity (Sled et al., 1998).

### MRI Image Analysis

The amygdala was automatically segmented using the multiple automatically generated templates (MAGeT) Brain pipeline (Chakravarty et al., 2013; Pipitone et al., 2014). Next, AGV were manually corrected (by RD) using a validated manual segmentation protocol (Pruessner et al., 2000), and a random subset were verified by an expert rater (JCP). Total Brain Volume (TBV) was obtained using the Brain Extraction based on non-local Segmentation Techniques (BEaST) method, which is based on non-local segmentation in a multi-resolution framework (Eskildsen et al., 2012). BEaST is designed to include cerebral spinal fluid (in the ventricles, cerebellar cistern, deep sulci, along surface of brain and brain stem), the brainstem, and cerebellar white and gray matter in the brain mask while excluding the skull, skin, fat, muscles, dura, eyes, bone, exterior blood vessels, and exterior nerves. After the BEaST masks were automatically created, the labels underwent quality control and manual corrections. Amygdala volumes were corrected for TBV (AGV/TBV) to account for individual differences in brain volume. The AGV/TBV variables were used in all analyses, and are referred to as normalized AGV.

### Statistical Analyses

All analyses were carried out on boys and girls separately to account for the difference between male and female neurodevelopmental trajectories, and given the sex-specific effects reported in the literature (Buss et al., 2012; Favaro et al., 2015). First, descriptive analyses (mean, range, standard deviation) were conducted on outcome, predictor and control variables, and *t*-tests were run to compare boys' and girls' levels for each of those variables. Pearson zero-order correlations for normally distributed variables, as well as Spearman's rho non-parametric testing for variables violating normality were also conducted to test the association between all variables.

#### Prenatal Maternal Stress and Amygdala Volume (Regression Analyses)

To test the effects of PNMS on normalized AGV, the data for right and left normalized AGV were subjected to hierarchical regression analyses. Child handedness was controlled for in all analyses. Given the evidence that timing of exposure to the stressor might influence the response to PNMS, timing of exposure to the storm *in utero* was also included in the models regardless of its association with the brain, behavioral and cognitive outcomes. However, the mother's age at birth of child, pregnancy drinking and smoking habits, as well as her perinatal SES and number of life events, and concurrent psychological symptoms at 11½ years postpartum were used as covariates but kept only if they were correlated with brain outcomes. This method of selectively adding covariates to the models allowed for the conservation of power considering the limitations imposed by our sample size. The regression model steps were the same for right and left normalized AGV (the outcome variable): the covariates were entered first into the model (timing of exposure, then handedness, then covariates associated with the outcome sequentially), followed by objective hardship, subjective distress, and lastly, cognitive appraisal.

To assess the moderating effects of timing of exposure on the relationship between PNMS and right and left normalized AGV, interaction terms were added in the last step of the hierarchical regression, with either Objective hardship × Timing of exposure, Subjective distress × Timing of exposure or Cognitive appraisal × Timing of exposure as the interaction terms, entered in separate analyses.

In order to determine the confounding effect of postnatal environment on the effect of PNMS, concurrent SES at 11½ years postpartum, as well as postnatal maternal life events, were then added to the models if they were significantly correlated with AGV. Even though concurrent maternal psychological symptoms are postnatal, this variable was included in all models if significant, to address a potential responder bias issue.

Given the sample size, the equations were trimmed of any non-significant variables (*p* > 0.1) that were forced into the equation except for handedness, objective hardship, timing of exposure to the storm, and any PNMS variable included in the interaction term. To control for the number of moderation analyses, the Bonferroni correction was applied to the interaction term *p*-values.

#### Amygdala Volume and Behavioral Outcomes (Partial Correlations)

To test the effects of left or right AGV on internalizing and externalizing problems partial correlations were run, controlling for covariates that were significantly associated with the behavioral outcomes. As per the regression analyses, handedness and timing of exposure to the storm were always included as covariates, and analyses were run with and without postnatal measures to determine their confounding effect.

#### Mediating Effect of Amygdala Volumes on Association Between PNMS and Behavioral Problems

To test the extent to which changes in normalized AGV explained the effect of PNMS on behavior, simple mediation or moderated mediation analyses were used. Because the indirect effect is more likely to be significant if the two paths forming it are strongly associated, the mediation or moderated mediation was only tested when both paths of the indirect effect (path 1: PNMS or PNMS by timing interaction to normalized AGV from the regressions, and path 2: normalized AGV to behavior from the partial correlations) were significant or marginally significant. Covariates that were significantly correlated with either normalized AGV or the behavioral outcome were entered in the model, and then trimmed out if not-significant in the final model. Again, the models were run with and without postnatal measures.

For the moderation analyses, the SPSS PROCESS macro (Hayes and Preacher, 2013) was used to run multiple linear regressions and probe the interaction. For the mediation and moderated mediation analyses, PROCESS uses bootstrapping with 20,000 resamplings to calculate the 95% confidence interval for the indirect effect. All correlation and main effect analyses were set to *p* < 0.05. Analyses were conducted using SPSS 20.0.

## Results

Descriptive statistics for outcome, predictor, and control variables are presented for boys and girls separately in Table 1. There was a strong tendency for boys to have longer gestational ages at birth (*p* = 0.054 and to have higher internalizing problem scores (*p* = 0.059). There was also a weak tendency for mothers of boys to have lower concurrent SES (*p* = 0.091) and to have experienced more postnatal major life events (0.092). Boys and girls did not significantly differ on any other variable. Non-significant analyses are presented in Supplementary Tables 1, 2.

**Table 1:** Descriptive statistics of variables, and results of *t*-test for sex differences.

	**Boys (*****n*** **=** **31)**		**Girls (*****n*** **=** **32)**		***p***	***Cohen's d***
**Variables**	**Mean**	**SD**	**Mean**	**SD**		
Right normalized AGV	0.070	0.007	0.072	0.008	0.274	0.278
Left normalized AGV	0.072	0.007	0.072	0.007	0.922	0.025
Objective stress storm32	11.839	4.810	11.031	4.816	0.508	0.168
Subjective stress IES-R	12.032	13.824	8.650	8.870	0.472	0.182
Number of days of pregnancy when ice storm happened (timing of PNMS)	78.450	102.849	94.590	103.788	0.538	0.156
Gestational age at birth (weeks)	40.226	0.976	39.754	0.927	0.054	0.495
Maternal psychological symptoms GHQ-28	0.106^b^	0.155	0.060^a^	0.115	0.192	0.338
Socioeconomic status (SES) Hollingshead scale (perinatal)	29.839	12.718	27.094	12.105	0.384	0.221
Socioeconomic status (SES) Hollingshead scale (concurrent)	34.31^c^	15.05	27.67^b^	14.60	0.091	0.448
Number of cigarettes/day	2.419	5.622	1.750	4.930	0.617	0.127
Number of glasses of alcohol/week	0.047	0.184	0.127	0.421	0.330	0.246
Maternal life events (perinatal)	6.516	4.098	5.468	2.961	0.248	0.294
Maternal life events (postnatal)	1.29^b^	0.89	0.9470	0.661	0.092	0.440
Obstetric complications	4.81	3.02	3.970	2.192	0.211	0.319
Maternal age at birth	29.000	5.320	30.196	4.311	0.330	0.247
Internalizing problems CBCL (T-scores)	54.73^b^	10.106	49.47	11.376	0.059	0.488
Externalizing problems CBCL (T-scores)	48.03^b^	8.732	45.81	8.716	0.320	0.255

*Lower SES scores represent higher SES*.

a *N = 31*.

b *N = 30*.

c *N = 29*.

*AGV, Amygdala volume; IES-R, Impact of Event Scale-Revised; PNMS, Prenatal maternal stress; GHQ, General Health Questionnaire; CBCL, Achenbach Child Behavior Checklist*.

### Boys

#### Prenatal Stress and Amygdala Volume (Regression Analyses)

As shown in Table 2, perinatal SES was correlated with left and right normalized AGV, thus, this variable was entered in both regression models. Additionally, concurrent SES and perinatal maternal life events were also correlated with left and right normalized AGV, and maternal psychological symptoms were associated with left normalized AGV, thus, those variables were entered accordingly into the two regression models adjusted for postnatal measures.

**Table 2:** Pearson's and Spearman's rho correlation coefficients between all variables split by sex.

	**1**	**2**	**3**	**4**	**5**	**6**	**7**	**8^b^**	**9^b^**	**10^b^**	**11^b^**	**12^b^**	**13^b^**	**14^b^**	**15^b^**	**16**	**17**	**18**
1- Right normalized AGV^a^	–	0.796^**^	0.055	0.045	0.144	0.256	−0.354^†^	0.243	−0.408^*^	−0.425^*^	−0.204	0.072	0.118	0.410^*^	−0.131	–	0.251	0.432^*^
2- Left normalized AGV^a^	0.765^**^	–	0.089	0.135	0.077	0.203	−0.322^†^	0.434^*^	−0.355^*^	−0.445^*^	−0.288	0.182	0.238	0.477^**^	−0.266	–	0.330^†^	0.457^*^
3- Objective hardship^a^	0.087	0.146	–	0.373^*^	−0.406^*^	−0.108	0.198	0.409^*^	−0.206	−0.135	0.012	−0.278	0.060	0.197	−0.106	–	0.053	0.133
4- Subjective distress^a^	0.229	0.266	0.212	–	−0.140	−0.066	0.016	0.269	0.263	0.264	0.158	−0.034	0.330^†^	0.277	0.172	–	0.126	0.124
5- Cognitive appraisal	−0.207	−0.216	−0.303^†^	−0.021	–	0.286	−0.196	0.059	0.045	0.149	0.135	0.074	0.032	0.282	−0.189	–	0.072	0.223
6- Timing of PNMS^a^	0.441^*^	0.285	−0.259	0.000	−0.184	–	−0.087	0.095	−0.189	−0.180	−0.048	−0.031	−0.061	0.197	−0.128	–	0.079	−0.097
7- Gestational age at birth^a^	−0.008	−0.155	0.037	−0.243	0.265	0.074	–	0.033	−0.240	−0.326^†^	0.116	−0.066	0.048	−0.290	0.159	–	0.153	−0.154
8- Maternal psychological, GHQ^b^	−0.127	−0.063	0.001	0.373^*^	0.265	−0.130	−0.286	–	−0.243	−0.191	0.031	0.112	0.429^*^	0.634^**^	−0.109	–	0.529^**^	0.390^*^
9- Socioeconomic status (perinatal)^b^	0.310^†^	0.209	0.303^†^	0.379^*^	−0.084	0.091	0.076	0.157	–	0.843^**^	0.353^†^	−0.003	−0.272	−0.146	0.079	–	−0.261	−0.129
10- Socioeconomic status (concurrent)^b^	0.280	0.193	0.412^*^	0.604^**^	−0.023	−0.037	0.019	0.198	0.883^**^	–	0.324^†^	−0.002	−0.108	0.051	0.293	–	−0.306	−0.075
11- Smoking^b^	0.176	0.150	0.213	0.400^*^	0.111	−0.232	−0.300^†^	0.109	0.327^†^	0.524^**^	–	0.032	−0.135	−0.130	0.413^*^	–	−0.073	−0.274
12- Alcohol usage^b^	−0.100	−0.185	0.031	−0.175	−0.324^†^	−0.148	−0.324^†^	−0.018	−0.031	−0.106	0.105	–	0.195	0.087	0.108	–	0.397^*^	0.391^*^
13- Maternal life events (perinatal)^b^	0.090	0.171	−0.045	0.289	−0.170	0.291	−0.260	0.101	0.049	0.051	0.131	−0.185	–	0.428^*^	0.132	–	0.326^†^	0.269
14-Maternal life events (postnatal)^b^	−0.171	0.074	0.264	0.322^†^	−0.079	−0.341^†^	−0.004	0.393^*^	0.007	0.156	−0.107	−0.239	0.224	–	0.055	–	0.394^*^	0.389^*^
15-Obstetric complications^b^	−0.036	−0.166	0.028	−0.011	0.136	−0.093	0.183	0.158	0.008	0.143	0.236	−0.020	−0.269	−0.049	–	–	0.050	−0.154
16- Maternal age at birth^a^	−0.107	−0.246	−0.285	0.137	0.072	−0.046	−0.027	−0.006	−0.199	−0.134	0.059	0.049	−0.200	0.007	0.307^†^	–	0.207	0.109
17- Internalizing problems^a^	0.116	0.092	−0.039	0.291	−0.051	−0.048	−0.260	0.189	0.107	0.236	−0.036	−0.120	0.131	0.496^**^	0.134	0.048	–	0.583^**^
18- Externalizing problems^a^	0.403^*^	0.378^*^	0.070	0.396^*^	−0.174	−0.045	−0.215	0.215	0.150	0.281	0.210	−0.009	0.350^*^	0.378^*^	0.151	−0.254	0.636^**^	–

*Correlations for boys (n = 31) are presented in the top right, and for girls (n = 32) in the bottom left. Lower socioeconomic status (SES) scores represent higher SES. AGV, Amygdala volume; PNMS, Prenatal Maternal Stress; GHQ, General Health Questionnaire*.

a *Pearson*.

b *Spearman*.

† *p < 0.1*,

* *p < 0.05*,

** *p < 0.01*.

##### Right amygdala volume

In boys, the results of the hierarchical regressions for right normalized AGV are presented in Table 3a. In the first step, timing explained 6.5% (*p* = 0.165), in the second step handedness explained an additional 2.7% (*p* = 0.373) and in the third step, SES explained an additional 13.0% (*p* = 0.043) of the variance in normalized AGV, such that higher SES significantly predicted larger AGV. In the fourth step, mothers' objective hardship explained an additional 0.3% (*p* = 0.755) and in the fifth step subjective distress explained an additional 3.9% (*p* = 0.207) of the variance in the child's right normalized AGV. Finally, the subjective distress × timing interaction term explained a statistically significant increase of 12.9% (*p* = 0.032) of the variance. Since no significant main or interaction effects were found with cognitive appraisal, it was left out of the final analyses. The full model explained 40.2% of the variance of the boys' right normalized AGV.

**Table 3:** Summary of hierarchical regression analyses for (a) the normalized right amygdala volume in boys and for (b) the normalized right and (c) left amygdala volume in girls at 11 years of age.

**Predictor variables**	**β**	***B***	***SE of B***	***R***	***R^2^***	***ΔR^2^***	***F***	***ΔF***
**(A) RIGHT NORMALIZED AGV—BOYS**								
Step 1				0.256	0.065		2.027	
Timing	0.256	1.76E-05	1.20E-05					
Step 2				0.303	0.092	0.027	1.417	0.820
Timing	0.294	2.02E-05	1.30E-05					
Preferred hand	0.167	0.005	0.005					
Step 3				0.471	0.222	0.130	2.562^†^	4.498^*^
Timing	0.258	1.78E-05	1.20E-05					
Preferred hand	0.126	0.004	0.005					
SES (perinatal)	−0.363^*^	−2.02E-04^*^	9.50E-05					
Step 4				0.474	0.225	0.003	1.882	0.099
Timing	0.255	1.76E-05	1.20E-05					
Preferred hand	0.145	0.004	0.005					
SES (perinatal)	−0.376^*^	−2.10E-04^*^	1.00E-04					
Objective stress	−0.060	−8.78E-05	2.79E-04					
Step 5				0.523	0.273	0.049	1.880	1.677
Timing	0.257	1.77E-05	1.20E-05					
Preferred hand	0.173	0.005	0.005					
SES (perinatal)	−0.480^*^	−2.68E-04^*^	1.08E-04					
Objective stress	−0.193	−2.84E-04	3.14E-04					
Subjective stress	0.263	0.002	0.001					
Step 6				0.634	0.402	0.129	2.687^*^	5.159^†^
Timing	0.095	−4.40E-05	2.90E-05					
Preferred hand	0.096	0.003	0.005					
SES (perinatal)	−0.526^*^	−2.93E-04^*^	1.01E-04					
Objective stress	−0.049	−7.27E-05	3.05E-04					
Subjective stress	0.072	−0.002	0.002					
Subjective stress^*^ timing	0.443^†^	2.57E-05^†^	1.10E-05					
**Adjusted for postnatal life events**				0.705	0.497	0.095	3.100^*^	4.057^†^
Timing	−0.074	−3.16E-05	2.88E-05					
Preferred hand	0.235	0.007	0.005					
SES (perinatal)	−0.444^*^	−2.47E-04^*^	1.00E-04					
Objective stress	−0.141	−2.09E-04	3.03E-04					
Subjective stress	0.028	−0.001	0.002					
Subjective stress^*^ timing	0.329	1.88E-05	1.13E-05					
Postnatal life events	0.368^†^	0.003^†^	0.001					
**(B) RIGHT NORMALIZED AGV—GIRLS**								
Step 1				0.441	0.194		7.227^*^	
Timing	0.441^*^	3.19E-05^*^	1.20E-05					
Step 2				0.463	0.214	0.020	3.954^*^	0.742
Timing	0.443^*^	3.21E-05^*^	1.20E-05					
Preferred hand	0.142	0.004	0.004					
Step 3				0.543	0.295	0.081	3.904^*^	3.202^†^
Timing	0.528^**^	3.82E-05^**^	1.20E-05					
Preferred hand	0.259	0.007	0.004					
Objective stress	0.318^†^	4.96E-04^†^	2.77E-04					
**(C) LEFT NORMALIZED AGV—GIRLS**								
Step 1				0.285	0.081		2.648	
Timing	0.285	1.82E-5	1.10E-05					
Step 2				0.358	0.128	0.047	2.137	1.575
Timing	0.289	1.84E-5	1.10E-05					
Preferred hand	0.218	0.005	0.004					
Step 3				0.491	0.241	0.113	2.969^*^	4.168^†^
Timing	0.389^*^	2.48E-5^*^	1.10E-05					
Preferred hand	0.357^†^	0.008^†^	0.004					
Objective stress	0.376^†^	0.001^†^	2.54E-04					

*AGV, amygdala volume*.

† *p < 0.10*.

* *p < 0.05 (2-tailed)*.

** *p < 0.01 (2 tailed)*.

*Lower SES scores represent higher SES*.

In (a) Bonferroni corrected on interaction p-value: ^†^p < 0.033. *p < 0.0167 (2-tailed).

**Figure 1:**
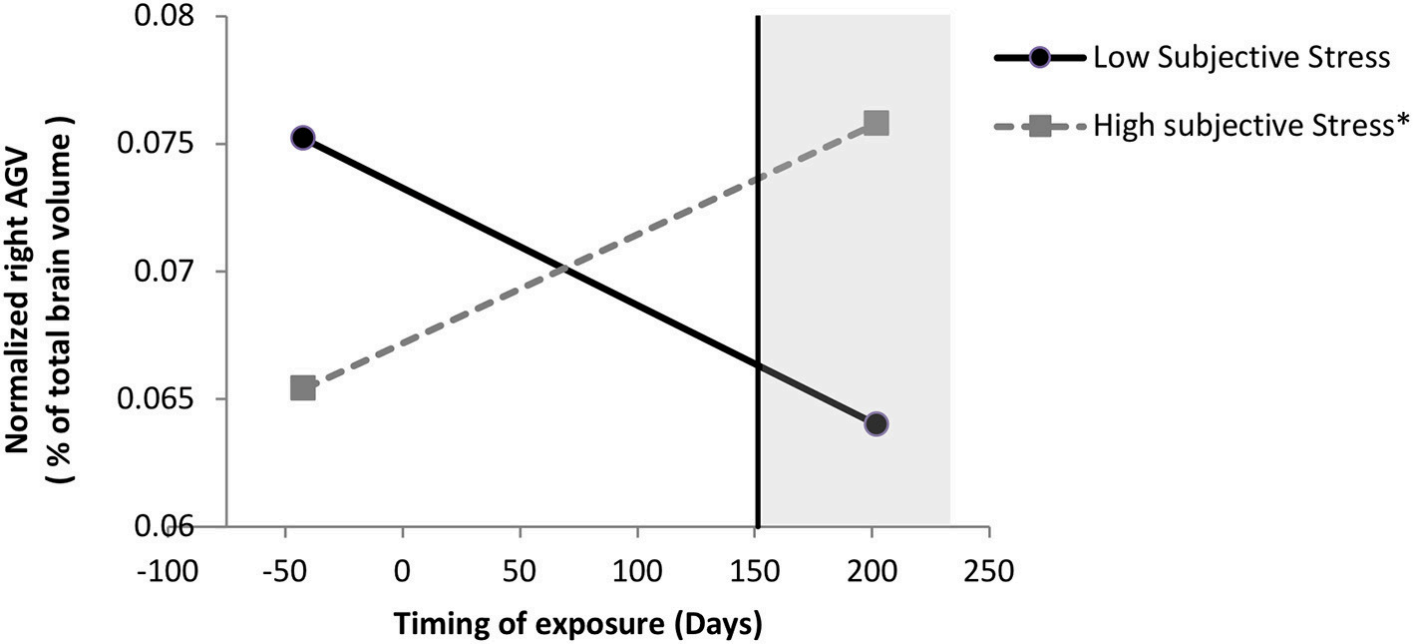
Moderation of subjective stress' effect on normalized right AGV by timing of exposure in boys. Low and high stress lines are represented at the 10 and 90th sample percentile of subjective distress (measured with the Impact of Events Scale-Revised) respectively, which are at a log-transformed level of 0 and 3.33 (0 and 26.94 in original scale), respectively. Following a significant interaction between subjective stress and timing, probing the interaction revealed that when mothers were exposed to the storm from day 157 (week 22.42) onwards, the greater their mothers' subjective stress, the larger their right normalized AGV; the region of significance is represented by the vertical line. When mothers were exposed to the storm before day 157, which includes the mothers exposed to the storm during preconception, there was no significant effect of subjective stress on right normalized AGV. Additionally, when subjective stress scores were equal to or greater than a log value of 2.64 (original subjective stress scale 13.01), there was a significant (*p* < 0.05) effect of timing on right normalized AGV; for these boys, the later they were exposed to the storm in gestation, the larger the right normalized AGV; for these boys, the later they were exposed to the storm in gestation, the larger the right normalized AGV. *p < 0.01.

We probed this interaction and found, as illustrated in Figure 1, that when the boys were exposed to the ice storm from pregnancy day 157 (week 22.42) onwards, there was a significant positive association between subjective distress and right normalized AGV: for boys exposed to the ice storm after pregnancy day 157, the greater their mothers' subjective distress, the larger the right normalized AGV. When the boys were exposed to the storm before pregnancy day 157, there was no significant effect of subjective distress on right normalized AGV. Additionally, when subjective distress scores were equal to or greater than a log value of 2.64 (original subjective stress scale 13.01), there was a significant (*p* < 0.05) effect of timing on right normalized AGV; for these boys, the later they were exposed to the ice storm in gestation, the larger the right normalized AGV.

When adjusting the model for postnatal measures, concurrent SES was no longer significant, so it was trimmed out of the model. Postnatal life events became marginally significant (*p* = 0.056), such that more yearly life events was associated with larger normalized AGV. However, adjusting for number of yearly postnatal life events, the subjective stress x timing interaction was no longer significant.

##### Left amygdala volume

For the left normalized AGV in boys, the hierarchical regression models revealed no significant main effects of PNMS measures, and no significant PNMS-by-timing interactions (data not shown).

#### Amygdala Volume and Behavioral Outcomes (Partial Correlations)

When testing the second path of the indirect effect we found that right and left normalized AGV in boys were associated with externalizing, but not internalizing, problems when controlling for timing of exposure, handedness, maternal psychological functioning, and maternal alcohol usage during pregnancy (Table 4). However, when adjusting for number of yearly postnatal maternal life events, the effect of normalized right AGV on externalizing became marginal.

**Table 4:** Partial correlation coefficients between amygdala volume (AGV) and behavioral problems controlling for handedness, timing of exposure to the storm and additional covariates (see footnotes).

**Sex child**	**Variables**	**Internalizing problems**	**Externalizing problems**
**PERINATAL MODEL**			
Boys	Normalized right AGV	0.167^a^^,^ ^b^	0.452^*^^a^^,^^b^
	Normalized left AGV	0.154^a^^,^^b^	0.412^*^^a^^,^^b^
Girls	Normalized right AGV	0.177	0.561^**^^c^
	Normalized left AGV	0.144	0.384^*^^c^
**ADJUSTING FOR POSTNATAL LIFE EVENTS**			
Boys	Normalized right AGV	0.082^a^^,^^b^^,^^d^	0.355^†^^a^^,^^b^^,^^d^
	Normalized left AGV	0.075^a^^,^^b^^,^^d^	0.313^*^^a^^,^^b^^,^^d^
Girls	Normalized right AGV	0.203	0.568^**^^c^
	Normalized left AGV	0.074	0.344^*^^c^

† *p < 0.10*.

* *p < 0.05 (2-tailed)*.

** *p < 0.01 (2-tailed)*.

a *Glasses of alcohol per week*.

b *Maternal psychological functioning (concurrent)*.

c *Maternal life events (perinatal)*.

d *Maternal life events (postnatal)*.

#### Mediating Effect of Amygdala Volumes on Association Between PNMS and Externalizing Problems

Because a significant subjective distress by timing interaction effect was obtained on right normalized AGV, and that partial correlations showed that right normalized AGV was significantly associated with externalizing problems, a moderated mediation effect was tested in boys, moderating the first path by timing of exposure, and adjusting the model for handedness, SES, maternal psychological functioning, and maternal alcohol usage. However, since neither path in the model showed a significant effect of maternal psychological functioning, this covariate was removed from the final model. The results indicated a significant moderated mediation effect (index of moderated mediation = 0.0135 and 95% confidence interval = [0.0006; 0.0369]), such that the mediation effect from higher subjective distress to more externalizing problems via larger right AGV was only significant for boys exposed on gestational day 212 (30.29 weeks) or later. However, since the moderation effect was no longer significant when adjusting for number of yearly postnatal life events, the mediation adjusting for postnatal measures was not tested.

### Girls

#### Prenatal Stress and Amygdala Volume (Regression Analyses)

As shown in Table 2, no covariates were correlated with normalized AGV in girls, so no additional variables were added to the models.

##### Right amygdala volume

In girls, the results of the hierarchical regressions for right normalized AGV are presented in Table 3b. In the first step, timing explained 19.4% (*p* = 0.012), such that later exposure was significantly associated with larger right normalized AGV. In the second step, handedness explained 2.0% (*p* = 0.396) of the variance in right normalized AGV. In the last step, mothers' objective hardship explained an additional 8.1% (*p* = 0.084) of the variance in the girls' right normalized AGV: higher objective hardship was related to a larger right normalized AGV. Neither subjective distress, cognitive appraisal, nor any of the interaction terms were significantly related to the girls' right normalized AGV. The full model explained a total of 29.5% of the variance.

##### Left amygdala volume

Results of the hierarchical regression for left normalized AGV in girls are presented in Table 3c. In the first step, timing explained 8.1% (*p* = 0.114), and in the second step, handedness explained an additional 4.7% (*p* = 0.220) of the variance in left normalized AGV. In the last step, mothers' objective hardship explained an additional 11.3% (*p* = 0.051) of the variance: higher objective hardship was related to a larger left normalized AGV. The main effects of mothers' subjective distress and cognitive appraisal were not significantly related to the girls' left normalized AGV so they were left out of the model. None of the interaction terms were significantly related to the girls' left normalized AGV. The final model explained 24.1% of the variance in the girls' left normalized AGV.

#### Amygdala Volume and Behavioral Outcomes (Partial Correlations)

When testing the second path of the indirect effect we found that larger right normalized AGV predicted more externalizing problems in girls when controlling for timing, handedness, and maternal life events (Table 4). There was no effect on internalizing problems when controlling for timing and handedness. Results were very similar when adjusting for postnatal measures.

#### Mediating Effect of Amygdala Volumes on Association Between PNMS and Externalizing Problems

Because objective hardship was marginally associated with left and right normalized AGV, which were associated with externalizing problems, we tested for mediation effects, adjusting for timing of exposure, handedness, and maternal life events. However, in the model with left normalized AGV, neither path showed a significant maternal life events effect, so this covariate was removed from the final model. The analyses revealed that more objective hardship predicted larger left normalized AGV which in turn was associated with more externalizing problems (indirect effect = 0.286 and 95% confidence interval [0.0166; 0.9643]). The same mediation effect was also observed for the right normalized AGV (effect: 0.3831 and confidence interval [0.0106; 1.2727]). When adjusting for postnatal measures, the yearly number of postnatal life events was no longer significant, so it was trimmed out of the models. As such, the mediations through left and right AGV remain significant.

## Discussion

The first aim of this study was to determine whether there is an association between disaster-related PNMS and normalized AGV in boys and girls, and to investigate the extent to which timing of the ice storm *in utero* moderates the effects of PNMS on AGV. The second aim was to determine the extent to which AGV mediated the effects of disaster-related PNMS on child behavioral outcomes. By using a natural disaster as the source of stress, our method included the ability to test the relative contribution of three aspects of pregnant women's disaster-related PNMS experience (i.e., objective hardship, subjective distress, and cognitive appraisal) on AGV and on behavioral functioning of their children. Given the sudden onset of the ice storm, we were also able to test, and control for, the timing of *in utero* maternal stress exposure. Because boys and girls respond to PNMS differently, and because previous research has shown a sex-specific effect of maternal cortisol on AGV (Buss et al., 2012), and on internalizing/externalizing symptoms (reviewed in Glover and Hill, 2012) we tested our hypotheses in boys and girls separately. Our results suggest that there is a complex relationship between the predictors (the aspects of maternal stress experienced during pregnancy, and timing) and AGV in exposed boys and girls at 11½ years of age.

### In Boys, Exposure to Higher Subjective PNMS in the Second Half of Pregnancy Is Associated With Larger Right Amygdala Volumes Which Predict More Externalizing Problems

Our first hypothesis was that higher levels of maternal objective hardship and/or subjective distress, and/or a negative cognitive appraisal of the crisis would explain variance in child AGV as normalized by total brain volume. No significant direct associations between our disaster-related PNMS measures and normalized AGV in boys were detected. From our correlational analyses we found that higher SES was associated with larger AGV, and when controlling for SES and objective PNMS exposure, an interaction between subjective PNMS and timing of exposure emerged. When exposure to the ice storm occurred at or after gestational week 22, higher maternal subjective distress predicted larger normalized right AGV. When subjective PNMS was >13 (which is not very severe), the later the exposure, the larger the AGV, such that AGV in those exposed earlier were below the sample's average while AGV in those exposed later were above the sample's average (Figure 1). The model explained 40% of the variance in normalized right AGV, with 13% attributed to SES, and 13% to the interaction between subjective PNMS and timing. Interestingly, when adjusting the regression model for postnatal measures, concurrent SES was trimmed out of the model because it was no longer significant, and postnatal life events became marginally significant (*p* = 0.056), such that more yearly postnatal life events was associated with larger normalized AGV. However, adjusting for number of yearly postnatal life events, the interaction between subjective stress and timing was no longer significant. This suggests that maternal reported postnatal life events are an important factor contributing to right normalized amygdala volume in boys. Larger studies that specifically address the influences of prenatal factors, timing of PNMS exposure, and of postnatal life events directly related to the child are needed to better understand the influence of these factors on AGV in boys.

These data add to a growing body of literature showing that the amygdala is vulnerable to early life adverse experiences and prenatal factors, and suggest that the timing of exposure may be important. Maternal cortisol levels and prenatal maternal depression have been associated with structural and functional changes in the amygdala, and some have shown that the effects are specific to girls (e.g., Favaro et al., 2015; Wen et al., 2017; Soe et al., 2018). Limitations from those studies include, in the maternal depression studies (Lupien et al., 2011), the inability to test for timing effects, and in the maternal cortisol study (Buss et al., 2012), the lack of an independent maternal stressor. Our study suggests that the pregnant mother's subjective distress in response to a sudden onset stressor from mid-gestation onward is associated with larger AGV in boys. Furthermore, and consistent with previous studies (Buss et al., 2012; Rifkin-Graboi et al., 2013), the right amygdala seems particularly vulnerable to the effects of prenatal factors and early life adverse experiences. Our findings suggest that structural changes in the amygdala in boys may be particularly sensitive to maternal subjective distress experienced from a natural disaster, as of the 22nd week of gestation. The direction of our effect is consistent with that of Buss et al. (2012), who reported that higher levels of maternal cortisol levels in healthy mothers during pregnancy were associated with larger AGV at 7 years of age (albeit the effect was found in girls, but not boys), however their effect was detected earlier, at 15 weeks of gestation. Fetal cortisol is produced throughout the second trimester (weeks 12–20) (Johnston et al., 2018), with no difference between the sexes, and the fetal adrenal gland begins to secrete cortisol at increased levels as of 22 weeks gestation (Mesiano and Jaffe, 1997). One possibility explaining these sex-specific timing effects could involve sex differences in placental conversion of active maternal cortisol to inactive cortisone by 11B-hydroxysteroid dehydrogenase type 2 (11β-HSD2) (Clifton, 2010; Nugent and Bale, 2015). Another possibility may relate to amygdala circuitry, which expands from local to distal in the third trimester, developing connections with the frontal and temporal lobes before making connections with the contralateral amygdala (Scheinost et al., 2017). Thus, it is possible that in the face of maternal subjective distress, it is the summed contribution of both maternal and fetal cortisol levels that affects fetal male amygdala development and circuitry.

Prenatal factors can also differentially influence structural properties of the amygdala at different points in postnatal development. For example, in one rodent study that considered only males, mild prenatal environmental stressors (starting on gestational day 14/21) was associated with smaller lateral, basolateral, and central nuclei on postnatal day 25 (i.e., early pubertal period), but not at postnatal days 7, 45, or 60. Interestingly, in the basolateral amygdala, but not the lateral or central nuclei, prenatal stress was associated with a shorter nucleus at postnatal day 25, no difference on postnatal day 45, but longer nucleus on postnatal day 60 (Kraszpulski et al., 2009). Thus, PNMS may differentially affect structural properties of different amygdala nuclei throughout development. Although for rodents and humans, peak amygdala development occurs at different time points during gestation (in the second half of gestation in rats, and in the first trimester in humans, as reviewed in Charil et al., 2010), exposure to maternal perinatal stress or stress hormones can have a lasting impact on postnatal amygdala development in both species. Our data are consistent with those from studies of children who were initially raised in an impoverished institution as young infants, but who were then brought up in very high SES families: these children also had larger AGV in later life (Mehta et al., 2009; Tottenham and Sheridan, 2009). As such, the larger AGV we observe may be due to decreased cell proliferation and neuronal differentiation and increased gliogenesis due to stressors experienced during critical periods of perinatal development, as proposed by others (Salm et al., 2004; Kang et al., 2011; Buss et al., 2012). Although it is unclear how such structural changes may relate to externalizing problems, those changes may disrupt typical connectivity to other structures related to emotional regulation.

In boys, higher maternal subjective distress predicted higher externalizing scores through larger normalized right AGV, but only when the ice storm occurred at or after gestational week 30. This suggests that although maternal subjective distress can influence right AGV as of 22 weeks of gestation (as described above), the behavioral consequences of increased AGV are more likely to result from even later exposure (i.e., week 30). Whereas, there was no main effect of PNMS on internalizing or externalizing problems in boys, the partial correlations from both normalized left and right AGV predicted more externalizing problems, explaining 20% of the variance. Although the bivariate correlation suggested a tendency for left AGV to be associated with internalizing problems in boys, no association was detected from the partial correlations, suggesting that AGV was not associated with internalizing problems. Buss et al. (2012) did not observe associations between maternal cortisol and amygdala volume in boys, nor between maternal cortisol at any of the gestational ages and affective problems. Similarly, we did not find associations between PNMS and internalizing behaviors in our sample of boys. Yet our findings extend those of Buss et al. (2012) by showing that maternal subjective distress at a later period of gestation is associated with larger normalized AGV in 11½ year old boys, which in turn is associated with concurrent externalizing behaviors.

### In Girls, Normalized Amygdala Volumes Mediate the Association Between Objective PNMS and Externalizing Problems

Our first hypothesis was that higher levels of maternal objective hardship and/or subjective distress, and/or a negative cognitive appraisal of the crisis would explain variance in the child's normalized AGV. The regression analyses revealed that when controlling for timing and handedness, greater objective hardship tended to predict larger right and left normalized AGV. Although there was no moderating effect of timing, a main effect of timing was detected in the hierarchical regression, such that later timing of exposure was associated with larger right and left normalized AGV. This timing effect is in contrast to Buss et al. (2012) who reported that maternal cortisol levels in earlier pregnancy were associated with larger AGV in girls at 7 years of age. As mentioned in section In Boys, Exposure to Higher Subjective PNMS in the Second Half of Pregnancy Is Associated With Larger Right Amygdala Volumes Which Predict More Externalizing Problems, these contrasting findings can be due to differences in the age of the participants when AGV was measured.

Our second hypothesis was that normalized AGV would mediate the association between PNMS and behavioral problems. The partial correlations revealed that when controlling for timing, handedness and maternal life events, larger right and left AGV were associated with more externalizing problems. The mediation analysis revealed that objective hardship was associated with larger left and right AGV, which in turn was associated with more externalizing problems at 11½ years of age. The existing literature suggests that more externalizing problems are associated with smaller AGV in adults (Matthies et al., 2012; Bobes et al., 2013; Gopal et al., 2013). However, prior to the current study only one study examined the mediating role of AGV between prenatal factors and behavior in children. Buss et al. (2012), in a group of healthy mothers and children, reported that higher maternal cortisol levels during early pregnancy were associated with larger right AGV which in turn was associated with more concurrent internalizing behaviors in 7 year old girls. That study considered naturally varying maternal cortisol levels collected at discrete periods during pregnancy (i.e., weeks 15, 19, 25, 31, and 39). The present study has the advantage of considering how a sudden-onset external stressor, outside of the mother's control, affected child development when exposed at varying prenatal periods. Here, we report that factors outside of the mother's control (i.e., her objective hardship experienced from the ice storm), and separate from her subjective response, is associated with larger normalized AGV and concurrent externalizing behaviors at 11½ years of age. Together these findings show that maternal factors during the prenatal period are important in the developing child's brain and behaviors.

The lack of associations with internalizing problems is surprising. We have previously reported with this sample that objective hardship and subjective distress are associated with internalizing problems when controlling for sex (King et al., 2012), whereas our bivariate correlations here suggest that subjective distress is more strongly associated with internalizing (and externalizing) in girls than in boys. Prospective studies have shown that maternal anxiety is a particularly strong predictor of child anxiety, regardless of sex (reviewed in Glover and Hill, 2012). Although the amygdala is thought to be an important structure associated with internalizing and externalizing problems, the directionality of those effects are not clear, which may be due to factors such as timing of the stressor, or the age of the child at the time of the assessment (Tottenham and Sheridan, 2009), or sex. Whereas some studies find smaller AGV in children and adolescents with mixed anxiety disorders (e.g., Mueller et al., 2013; Strawn et al., 2015), others report larger AGV in children with higher parent-reported anxiety (Qin et al., 2014). Both larger and smaller volumes have been reported in pediatric anxiety/depression (De Bellis et al., 2000; Milham et al., 2005), but sub-clinical internalizing problems appear to be associated with smaller AGV in otherwise healthy individuals (Spampinato et al., 2009; Merz et al., 2018). Similarly, it is smaller AGV that appears to be associated with higher levels of aggression in typical children (Matthies et al., 2012; Thijssen et al., 2015). Although the direction of these associations are unclear, Merz et al. (2018) recently reported that associations between environmental factors (i.e., SES) and AGV may vary by age. Our current findings suggests that larger, not smaller, AGV at 11½ may be predictive of concurrent aggressive behaviors, but not internalizing behaviors, particularly following *in utero* exposure to maternal stressors. Future studies are needed to disentangle the complex relationship between prenatal maternal stress and/or cortisol levels and behavioral problems as mediated through AGV, as their interaction with sex of the child, timing of exposure, and timing of assessment.

### Sex-Specific Effects of the Maternal PNMS Experience on Child Outcomes

The various elements of the maternal stress experience (i.e., objective hardship, subjective distress, and cognitive appraisal) on the brain appear to be different for males and for females. We report that for girls, objective hardship seems to be the strongest predictor of AGV. For boys, subjective distress has some mild influence on AGV. Cognitive appraisal was not associated with AGV in neither sex. Our findings that only objective hardship, not subjective distress, was associated with externalizing behaviors via AGV in girls are surprising because they do not fit the conventional model of subjective distress and cortisol where more subjective distress during pregnancy causes an increase in maternal cortisol, which affects fetal development. This suggests that some distinct aspects of the objective hardship, perhaps unrelated to maternal cortisol levels, are influencing child prenatal development. Unfortunately, no other human studies have investigated the specific effect of objective maternal hardship on the offspring brain so it is difficult to discuss our findings in light of previous research. Interestingly, it was maternal subjective distress, and not objective hardship, in late pregnancy, that was associated with larger AGV and increased externalizing behaviors in boys. These sex-specific effects, and how they interact with timing of exposure *in utero*, warrant further investigation. In the only human PNMS study on adult brain development, young women were found to have morphometric and functional changes in gray matter density within both the right and left amygdalae (Favaro et al., 2015), but men were not included in that study. Unfortunately, not only was the assessment of PNMS in that study retrospective, it also combined varying sources of stressors such as interpersonal problems (separation from partner), severe health problems, death of a loved one, abuse, or exposure to a natural disaster (Favaro et al., 2015). The design of our prospective longitudinal study allows the objective nature of the stress exposure to be isolated from the subjective distress of the woman and from her cognitive appraisal of the event, and the current data show that these different aspects of the maternal stress experience matter. In summary, our findings are consistent with those of Favaro et al. (2015), in that both the left and right amygdala are affected by PNMS in women, and we attribute this finding to the objective hardship exposure.

Both maternal subjective distress and objective hardship have been associated with cognitive and behavioral outcomes in the child (Watson et al., 1999; Wadhwa et al., 2001; Gutteling et al., 2005; Rice et al., 2007; Chuang et al., 2011; Liu, 2011). Moreover, in our previous Project Ice Storm studies, objective hardship was strongly correlated with physical, physiological and cognitive measures (Laplante et al., 2004, 2007, 2008, 2016; King and Laplante, 2005; King et al., 2009, 2012). Also, it was reported that objective hardship was strongly associated with DNA methylation (Cao-Lei et al., 2015, 2016), insulin secretion (Dancause et al., 2013), cytokine production (Veru et al., 2015), and earlier age at menarche (Duchesne et al., 2017), suggesting that the mechanisms through which objective hardship affects offspring development may bypass the maternal HPA axis and use other pathways to influence development of these systems. Lastly, our present findings linking subjective and/or objective PNMS with externalizing problems are supported by previous Project Ice Storm findings with assessments done 4½, 5½, 6½, 8½, and 9½ and 11½ years of age (King et al., 2012; Nguyen et al., 2018). Importantly, the current results suggest that different aspects of disaster-related PNMS can have sex-specific effects on neurodevelopment, and in turn explain variance in psychopathological symptoms (though the average T-scores lie within the normal range, as can be seen in Table 1).

### Limitations

There are limitations to this work that need to be acknowledged. An important limitation regarding the sex specific effects is the sample size that limited our power to test the interaction with sex. However, based on the existing literature on sex-specific effects, we used statistical approaches that have been suggested as best practices in studies that were underpowered to test sex differences (i.e., sex disaggregation, Heidari et al., 2016; Day et al., 2017; Lee, 2018. Thus, our sex-specific analyses suggest that boys and girls are differentially affected by PNMS. We have reported the interactions in Supplementary Table 3, and note that the interaction between right normalized AGV and sex had the strongest effect size (*R*^2^ change), consistent with the sex difference in right AGV reported by Buss et al. (2012). Moreover, the effect size we report for the interaction term was strongest for objective hardship, suggesting that this aspect of the maternal stress experience may be a particularly important driver in a potential sex difference. Future studies could use these reports to ensure sufficient power to specifically address sex differences.

Another important limitation to this study is that we did not collect maternal cortisol at the time of the stressor. This is a logistical limitation as the ice storm was a sudden-onset natural disaster and, due to delays in obtaining ethics approval, data collection only began 5 months after the disaster. In addition, although we were able to control for maternal reported life events, we did not have a direct measure of child self-reported life stressors in childhood. We used an indirect measure of child life events by using maternal related postnatal life events. These appear to be an important factor related to normalized amygdala volume in boys, and warrants further investigation. Another limitation is that we did not collect information on pubertal stage during the assessment at 11½ years of age. However, age at menarche was collected in 23 female participants at the 13½ year assessment. The mean age at menarche was 12. Only 5 out of the 23 female participants had attained menarche by the time of the MRI scan at age 11½. When looking at a scatterplot of the PNMS-by-AGV correlation, none of those 5 participants were outliers. No association was found between AGV in girls at age 11½ and their age at menarche (Table 2). Nonetheless, having pubertal stage information for the full sample may have improved our understanding of the effect seen, and may have helped remove the potential confounding effect of pubertal hormone surges on the brain and behavior. The lack of an unexposed control group is another important limitation to this study. And finally, although it is standard to correct brain region volumes for total brain volumes, it is possible that the volumetric changes we observed in amygdala volumes may be influenced by volumetric differences in other brain structures.

### Strengths

Our study's main strength was the use of a sudden-onset, quasi-randomly distributed natural disaster as a stressor, rather than studying maternal psychological state or potentially non-independent life events. This allowed us to test for dose-response effects of PNMS on child neural and behavioral outcomes while reducing the influence of genetic factors. Moreover, we were able to assess the various elements of stress soon after the event occurred, thus providing us with a highly personalized, deep-level measure of the various stress levels. Additional strengths of our present study include its longitudinal prospective design with multiple assessment points between birth and childhood. This allowed us to include a number of maternal, familial, and postnatal child factors that may also influence child development, such as perinatal and concurrent SES, obstetric complications, gestational age at birth, maternal smoking and drinking habits during pregnancy, and maternal life events which were included as control variables in this study. Finally, segmentation of all child brain MRI was done with gold-standard manual segmentation. This may be especially important for the assessment of AGV because it has been suggested that automatic segmentation protocols are less reliable for smaller subcortical structures (Tae et al., 2008), including the amygdala (Schoemaker et al., 2016).

## Conclusion

Our present findings suggest that in boys, a mother's distress from a natural disaster, when experienced in the second half of pregnancy, can influence the development of her child's amygdala, which in turn mediates the association between subjective PNMS and externalizing behaviors when measured 11½ years later. In girls, the objective hardship experienced from a natural disaster predicted larger right and left normalized AGV, which in turn was associated with more externalizing behavior. This is, to the best of our knowledge, the first report linking PNMS with subsequent AGV and behavioral problems in childhood. Findings from the present study provide support for the hypothesis that susceptibility to behavioral problems may, in part, be programmed *in utero*, and that this effect may be mediated through the development of the amygdala. Furthermore, the study shows that exposure to a stressor during gestation exerts a lasting influence on child development. These results add to the growing awareness of the importance of the intrauterine environment and reveal a new pathway through which the maternal exposure to a stressor during pregnancy may affect the offspring, in a sex-specific manner.

## Data Availability

We haven't obtained consent (mothers) or assent (children) to make the data available in publically accessible repositories, but they are available on request to the corresponding author.

## Ethics Statement

This study was carried out in accordance with the recommendations of The Code of Ethics of the World Medical Association, and approved by the Douglas Mental Health University Institute Research Ethics Board. All subjects gave written informed consent or written informed assent for participants under the age of 18 years, in accordance with the Declaration of Helsinki.

## Author Contributions

SK designed and implemented Project Ice Storm. RD, DL, JP, and SK conceived of the current experiment. RD and RP ran the automated segmentation pipelines, which was overseen by MC. GE and RD ran the statistical analyses. RD did manual corrections of the amygdala and total brain volume segmentations derived from the automated segmentations, overseen by JP. SK, DL, MC, and JP provided intellectual contributions to the interpretation of data. RD interpreted the data and drafted an early version of the manuscript. SJ, SK, and DL provided intellectual contributions for the rationale, interpretation of the data, and prepared the final manuscript for submission.

### Conflict of Interest Statement

The authors declare that the research was conducted in the absence of any commercial or financial relationships that could be construed as a potential conflict of interest.
