# Supplementary material for: Larger Amygdala Volume Mediates the Association Between Prenatal Maternal Stress and Higher Levels of Externalizing Behaviors: Sex Specific Effects in Project Ice Storm
Source: Front Hum Neurosci. 2019 May 14;13:144. doi: 10.3389/fnhum.2019.00144 (PMC6528106; doi:10.3389/fnhum.2019.00144)
Supplement: Supplementary file 1 [file Table_1.docx]

Supplementary Table 1. Summary of hierarchical regression analyses for left amygdala volume in boys.

| **Predictor Variables** | ***β*** | ***B*** | ***SE of B*** | ***R*** | ***R^2^*** | ***∆R^2^*** | ***F*** | ***∆F*** |
| --- | --- | --- | --- | --- | --- | --- | --- | --- |
| **a) Right normalized AGV - Boys** | | | | | | | | |
| Step 1 |  |  |  | 0.203 | 0.041 |  | 1.244 |  |
| Timing | 0.203 | 1.47E-05 | 1.32E-05 |  |  |  |  |  |
| Step 2 |  |  |  | 0.220 | 0.049 | 0.007 | 0.715 | 0.220 |
| Timing | 0.223 | 1.62E-05 | 1.37E-05 |  |  |  |  |  |
| Preferred Hand | 0.089 | 0.003 | 0.006 |  |  |  |  |  |
| Step 3 |  |  |  | 0.403 | 0.162 | 0.114 | 1.741 | 3.658† |
| Timing | 0.190 | 1.38E-05 | 1.32E-05 |  |  |  |  |  |
| Preferred Hand | 0.050 | 0.002 | 0.005 |  |  |  |  |  |
| SES (perinatal) | -0.340† | -2.00E-04† | 1.04E-04 |  |  |  |  |  |
| Step 4 |  |  |  | 0.403 | 0.162 | 0.000 | 1.258 | 0.003 |
| Timing | 0.190 | 1.38E-05 | 1.35E-05 |  |  |  |  |  |
| Preferred Hand | 0.047 | 0.001 | 0.006 |  |  |  |  |  |
| SES (perinatal) | -0.337* | -1.98E-04* | 1.09E-04 |  |  |  |  |  |
| Objective stress | 0.011 | 1.69E-05 | 3.05E-04 |  |  |  |  |  |
| **Adjusted for Postnatal Life Events** | | |  | 0.715 | 0.511 | - | 4.807** | - |
| Timing | 0.035 | -2.54E-06 | 1.13E-05 |  |  |  |  |  |
| Preferred Hand | 0.197 | 0.006 | 0.005 |  |  |  |  |  |
| Objective stress | -0.127 | -1.97E-04 | 2.52E-04 |  |  |  |  |  |
| SES (Postnatal) | -0.456** | -.2.32E-04 | 7.73E-05 |  |  |  |  |  |
| Postnatal Life Events | 0.576** | .005† | .001 |  |  |  |  |  |
