# Supplementary material for: Larger Amygdala Volume Mediates the Association Between Prenatal Maternal Stress and Higher Levels of Externalizing Behaviors: Sex Specific Effects in Project Ice Storm
Source: Front Hum Neurosci. 2019 May 14;13:144. doi: 10.3389/fnhum.2019.00144 (PMC6528106; doi:10.3389/fnhum.2019.00144)
Supplement: Supplementary file 2 [file Table_2.docx]

Supplementary Table 2. Summary of regression analyses of moderation by timing of exposure for models unadjusted for and adjusted for postnatal measures.

| **Outcome** | **Predictor** | **Unadjusted model** | | | **Adjusted for postnatal measures** | | |
| --- | --- | --- | --- | --- | --- | --- | --- |
|  |  | **Model R²** | **Moderation**  **R² change** | **Moderation**  **p-value** | **Model R²** | **Moderation**  **R² change** | **Moderation**  **p-value** |
| **Boys** | | | | | | | |
| Right AGV | Objective Hardship | 0.290 | 0.066 | 0.140 | 0.455 | 0.034 | 0.243 |
| Left AGV | Objective Hardship | 0.247 | 0.085 | 0.106 | 0.518 | 0.007 | 0.582 |
| Right AGV | Subjective Distress | **0.402** | **0.129** | **0.032** | 0.497 | 0.063 | 0.111 |
| Left AGV | Subjective Distress | 0.306 | 0.064 | 0.151 | 0.526 | 0.000 | 0.919 |
| Right AGV | Cognitive Appraisal | 0.262 | 0.015 | 0.503 | 0.433 | 0.010 | 0.542 |
| Left AGV | Cognitive Appraisal | 0.209 | 0.052 | 0.230 | 0.546 | 0.031 | 0.255 |
| **Girls** | | | | | | | |
| Right AGV | Objective Hardship | 0.341 | 0.046 | 0.181 | - | - | - |
| Left AGV | Objective Hardship | 0.286 | 0.045 | 0.204 | - | - | - |
| Right AGV | Subjective Distress | 0.351 | 0.022 | 0.352 | - | - | - |
| Left AGV | Subjective Distress | 0.298 | 0.011 | 0.537 | - | - | - |
| Right AGV | Cognitive Appraisal | 0.313 | 0.017 | 0.425 | - | - | - |
| Left AGV | Cognitive Appraisal | 0.276 | 0.032 | 0.298 | - | - | - |
