# Supplementary material for: Larger Amygdala Volume Mediates the Association Between Prenatal Maternal Stress and Higher Levels of Externalizing Behaviors: Sex Specific Effects in Project Ice Storm
Source: Front Hum Neurosci. 2019 May 14;13:144. doi: 10.3389/fnhum.2019.00144 (PMC6528106; doi:10.3389/fnhum.2019.00144)
Supplement: Supplementary file 3 [file Table_3.docx]

| Outcome | Predictor | Model R² | Interaction ΔR² | Interaction p-value |
| --- | --- | --- | --- | --- |
| Right Normalized AGV^a^ | Objective Stress | 0.256 | 0.013 | 0.323 |
|  | Subjective Stress | 0.256 | 0.006 | 0.4946 |
|  | Cognitive Appraisal | 0.269 | 0.006 | 0.523 |
| Left Normalized AGV^b^ | Objective Stress | 0.311 | 0.008 | 0.421 |
|  | Subjective Stress | 0.305 | 0.001 | 0.787 |
|  | Cognitive Appraisal | 0.338 | 0.001 | 0.848 |

Supplementary Table 3. Summary of moderating effect of sex on the association between PNMS and normalized amygdala volumes.

a. Control Variables: Storm32, Handedness, Timing of exposure, Gestational age at birth.

b. Control Variables: Storm32, Handedness, Timing of exposure, Gestational age at birth, postnatal events.
